# Mapping emerging technologies in aged care: results from an in-depth online research

**DOI:** 10.1186/s12913-023-09513-5

**Published:** 2023-05-23

**Authors:** Annachiara Fasoli, Giorgia Beretta, Gabriella Pravettoni, Virginia Sanchini

**Affiliations:** 1 Department of Oncology and Hemato-Oncology, University of Milan, Milan, 20122 MI Italy; 2 Psycho-Oncology Division, European Institute of Oncology, Milan, 20141 MI Italy; 3 Department of Public Health and Primary Care, Centre for Biomedical Ethics and Law, KU Leuven, Leuven, 3000 Belgium

**Keywords:** Emerging technologies, Older adults, Aged care, Ethical issues, Review, Active aging

## Abstract

**Background:**

Emerging Technologies (ETs) have recently acquired great relevance in elderly care. The exceptional experience with SARS-CoV-2 pandemic has emphasized the usefulness of ETs in the assistance and remote monitoring of older adults. Technological devices have also contributed to the preservation of social interactions, thus reducing isolation and loneliness. The general purpose of this work is to provide a comprehensive and updated overview of the technologies currently employed in elderly care. This objective was achieved firstly, by mapping and classifying the ETs currently available on the market and, secondly, by assessing the impact of such ETs on elderly care, exploring the ethical values promoted, as well as potential ethical threats.

**Methods:**

An in-depth search was carried out on Google search engine, by using specific keywords (e.g. technology, monitoring techniques, ambient intelligence; elderly, older adults; care and assistance). Three hundred and twenty-eight technologies were originally identified. Then, based on a predetermined set of inclusion-exclusion criteria, two hundreds and twenty-two technologies were selected.

**Results:**

A comprehensive database was elaborated, where the two hundred and twenty-two ETs selected were classified as follows: category; developmental stage; companies and/or partners; functions; location of development; time of development; impact on elderly care; target; website. From an in-depth qualitative analysis, some ethically-related contents and themes emerged, namely: questions related to safety, independence and active aging, connectedness, empowerment and dignity, cost and efficiency. Although not reported by developers, a close analysis of website contents highlights that positive values are often associated with potential risks, notably privacy threats, deception, dehumanization of care.

**Conclusions:**

Research findings may ultimately lead to a better understanding of the impact of ETs on elderly people.

**Supplementary Information:**

The online version contains supplementary material available at 10.1186/s12913-023-09513-5.

## Introduction

### Background

In recent years, life expectancy has increased considerably, especially in developed countries [1]. Consequently, the rate of population aging is progressing rapidly and, as the portion of people over 65 years of age continues to grow, demographic aging becomes one of the most serious challenges that health and social systems worldwide have to face [2, 3]. Traditional care models are struggling to meet the needs of this rapidly aging global population: there is a growing demand of healthcare services, a shortage of healthcare professionals and a huge burden is imposed on informal caregivers. Therefore, significant changes are required [4, 5].

Lately, technology has become more and more pervasive in our society and technological approaches have been introduced as a tool to ensure the provision of support in activities of daily living, acquiring great relevance also in the field of elderly care [3, p.S14]. A wide range of technological devices designed specifically for older people have appeared on the market, introducing new forms of assistance, and contributing to the construction of new models of long-term care [3, 6–8]. Most of these devices pertain to the category of Emerging Technologies (ETs). An ET may be defined as a “radically novel and relatively fast-growing technology characterised by a certain degree of coherence persisting over time and with the potential to exert a considerable impact on the socio-economic domain(s)” [9]. ETs are those technologies which feature, for instance, the use of Artificial Intelligence (AI), Internet of Things (IoT), big data, robotics and virtual reality. In the context of elderly care, the ETs which have been introduced are very diverse: wearable devices and environmental sensors, which constantly collect data for monitoring; assistive robots, which can help in daily activities and can keep older people company; virtual reality headsets, which allow the immersion in alternative settings for physical rehabilitation, cognitive exercises or entertainment. In this paper, from this point forward, the term ETs will be used to refer to this whole set of technologies exclusively developed for the assistance and care of the elderly.

The exceptional experience with SARS-CoV-2 pandemic and the implementation of containment measures brought these innovations in elderly care to light, showing, in particular, the usefulness of ETs in the assistance and remote monitoring of older adults. These tools contributed also to the preservation of seniors’ social interactions, allowing them to communicate with healthcare professionals, family and friends, reducing their isolation and loneliness [10]. ETs are proving to be transformative tools, promoting “active aging” [11], patient engagement [12] and allowing the so-called “aging-in-place” [13]. However, the impact of ETs on elderly care ought to be explored further, as it is not devoid of ambivalences and (especially ethical) concerns.

Indeed, in line with the diffusion of ETs in elderly care, interest in the research and study of these phenomena has also increased. A new field of research has appeared: *gerontechnology*, concerned with research combining technological advances and the study of ageing [2, p. 89]. At the academic level, a vast amount of literature has appeared addressing the subject. Some works have discussed these technologies from a conceptual and philosophical standpoint, exploring the ethical implications of (already or still to be implemented) ETs in the context of daily management and care of older adults [14–16]. Others have conducted qualitative and quantitative studies to understand the impact that ETs may have on the elder population [17]. Amongst them, some works have also explored the perspectives of seniors and caregivers on the desirable features of ETs and what they would expect from different technologies, in order to design devices targeted on their specific needs [18, 19].

However, to the best of our knowledge, there is no scholarly contribution based on the in-depth quantitative and qualitative content analysis of ETs’ websites, which combines a comprehensive mapping of the ETs designed for seniors available on the market with an ethical reflection over their impact on elderly care.

### Objectives

The present work is conceived with the intention of bridging the above-mentioned gap in the literature and its final aim is to assess the distribution and characterization of ETs and their impact on elderly care, in particular exploring “ethically-related contents” [20]. Its novelty lies in the investigation of technology developers’ perspective, as it emerges from ETs’ websites.

In order to achieve such objectives, this paper is based on the collection of information from ETs’ websites in a well-organized comprehensive database. This database constituted an indispensable tool for subsequent theoretical elaboration and ethical reflection. In our view, such a comprehensive database may also contribute to elderly care in a broader sense. This organized data collection may be the starting point for further analysis on these devices, investigating, for instance, market demands, monitoring the evolution of the industry and providing significant information for policy makers and managers of care to take decisions about resource allocation.

### Research questions

In line with the aforementioned objectives, the following research questions were formulated:

1. What types of ETs are currently available on the aged care market?
2. What are the ETs’ main functions?
3. What specific target were the ETs developed for?
4. Did COVID-19 pandemic influence the development and use of ETs in elderly care? And if so, how?
5. What are the ethically-related contents that emerge from the analysis of ETs’ websites?

The paper is structured as follows. First, in the Methodology section, we explain how the in-depth web search was carried out; we also provide indication related to data extraction and synthesis. Second, results of the research are presented, with a first part reporting general information about ETs retrieved, and a second part exploring in detail ethically-related themes and contents, emerged from website analysis. Finally, Discussion is devoted to the analysis and deep exploration of potential threats and solutions to critical issues previously identified.

## Methods

### Search strategy

In order to identify the ETs available on the market and in use either in nursing homes or in private homes, an in-depth research was conducted using Google search engine. The research was carried out in January 2022. Search strings were developed by combining three groups of keywords: Group A referred to the ETs, Group B concerned the target population, Group C related to the area of application. The keywords used for each group are shown in Table 1.

**Table 1:** Groups of keywords used to perform the research on Google search engine

Group	Keywords
Group A	Digital device(s), digital system(s), electronics, electronic device(s), electronic equipment, Technology, technologies, technological device(s)
Group B	Aged people, elderly, elders, older adults, older people, seniors
Group C	Assistance, care, cure, service, treatment

Examples of combinations of keywords as used in our search are reported in Table 2.

**Table 2:** Examples of combinations of keywords

a) b) c)	Assistance aged people digital devices Technology elderly care Electronic devices older adults treatment

Keywords were developed both in English and in Italian in order to identify and include also the ETs which are predominantly used and commercialised in Italy.

The Italian focus is justified on the basis that this research is part of a broader project, “ElderTech” (i.e. “Emerging Technologies and Vulnerabilities in Aged Care”, project number: 2020-1322), funded by an Italian foundation (Fondazione Cariplo), with both a national and international impact. As a result, this work charts ETs available internationally, with a specific focus on the Italian context. The search by keywords, combined with a snowballing process, identified a wide range of technologies. Information about each ET was gathered from one or more sources and organized in a database. Technologies’ websites were the main sources of information used. Then, the ETs retrieved were screened, based on a prespecified set of inclusion criteria (see below), in order to obtain a final list.

### Inclusion criteria

In selecting the ETs relevant for this review, authors used the following inclusion criteria: (i) the technologies are specifically developed for the elderly population; (ii) the field of application is aged care; (iii) information about the technologies can be retrieved from websites in English or Italian.

### Exclusion criteria

The following types of technologies were, instead, excluded from this review: (i) technologies targeted to the general population; (ii) technologies targeted to caregivers; (iii) technologies targeted to elders’ family members; (iv) the field of application is not aged care (broadly understood); (v) websites are not in English or Italian.

### Data extraction and synthesis

The first author (AF) performed the in-depth web search and identified a total of 328 ETs; then AF examined the results in relation to the inclusion/exclusion criteria reported above. The originally identified ETs underwent a selection process, manually carried out by AF, after which 222 technologies met the inclusion criteria and were included, whereas the remaining technologies were excluded (N = 106). The second (GB) and the last (VS) authors checked the final list of 222 ETs. The inter-rater reliability score was high, thanks to the clear definition of inclusion and exclusion criteria, which enabled the authors to identify the ETs of interest with a high degree of agreement. For the questionable ETs (2%, corresponding to 4 ETs), the first (AF) and the second (GB) authors discussed the candidate ET until agreement was reached.

After gathering all pertinent information from the websites and examining it, an offline archive consisting of a Microsoft Excel database, was created with the selected ETs.

Data extraction consisted in examining the websites linked to each technology, identifying all the information relevant to the previously stated research questions, and inserting such pieces of information in the database. As a result, the ETs in the database were classified according to the following characteristics:

- Category;
- Function;
- Target: autonomous seniors, semi-autonomous seniors, seniors with Mild Cognitive Impairment (MCI) and/or Dementia and/or Alzheimer’s disease;
- Time of development: before or during SARS-COV-2 pandemic;
- Additional information: developmental stage (ETs in the project stage or ETs already on the market); funding (parent company and/or partners); locations where ETs were developed.

Furthermore, a section of the database, in correspondence of each ET, was compiled with websites textual content which was deemed ethically relevant, as it referred to concepts and issues typically addressed by well-known ethical approaches (see below).

The final list of 222 ETs has then been thoroughly analyzed and a wide range of general findings have emerged. Drawing on the information collected about each technology, we elaborated comprehensive tables and descriptive graphs, which have been instrumental for presenting the results, quantifying their distribution and allowing a comparative evaluation. In performing website analysis, the first (AF) and the second (GB) authors used a double process of qualitative analysis. First, a *manifest content analysis* of the written content of the websites was carried out [21]. That means that the authors carefully analyzed and described the content of the websites by sticking to the literal meaning of the words [22, p.128]. Second, a *directed content analysis* was applied [23]. Themes and concepts stemming from well-known ethical approaches, i.e. relational care ethics, principlism and public health ethics, were applied to analyse website contents, as explained below in the second Results subsection.

In fact, the following results section is structured into two subsections. The first subsection presents the general results, providing data concerning categories, functions, target, and time of development of ETs, thus answering to research questions 1 to 4 and offering also some additional information on the ETs. The second subsection presents ethically-related themes and contents, emerged from the website analysis, providing some information about the ethical impact that ETs have on elderly care, thus providing an answer to research question 5.

## Results

### General results

#### Categories of ETs

In analyzing the results of our in-depth web search, we recognized that the ETs could be grouped into four main categories: Conventional Monitoring Techniques (CMTs), Unconventional Monitoring Techniques (UNMTs), Virtual Reality Technologies (VRTs), and Socially Assistive Robots (SARs).

CMTs refer to the set of techniques allowing a continuous observation of older adults (physiological and physical) condition performed through the use of traditional devices, such as the oximeter or the glucometer, integrated into or accompanied by a technological platform. CMTs are able to monitor older adults’ condition in their everyday lives and communicate relevant health and behavioural information in real time to health-care professionals or reference family members. They include the traditional devices used for telemedicine, to track physiological parameters or administer medicines.

UNMTs, instead, refer to the subcategory of monitoring techniques which perform their monitoring functions in a more pervasive manner. Examples of UNMTs are wearable devices – products controlled by electronic components and often equipped with GPS trackers, which are incorporated into clothing or worn on the body like accessories; but also Ambient Intelligence systems, defined as the sets of different physical environments (e.g. homes) interacting with people through computing devices, capturing pervasive information over the elder daily life.

The third category, VRTs, refers to the use of headsets that create 3D environments, in which the elder is fully immersed for therapeutic or entertainment purposes.

Finally, SARs represent the last frontier of aged care. SARs are defined as robots that provide assistance to older people, primarily through social or physical interaction. They can be used as companionship tools or to help in different tasks of the everyday life.

We classified each technology we found in one of these categories, and we observed the distribution which follows. The comprehensive list of ETs included in the review with the specification of category/function is reported in supplementary information (see Additional File 1).

Monitoring Techniques (see Table 3) constitute a significant majority of all ETs. Among the technologies belonging to this category, some are CMTs, but most of them are UNMTs. Examples of technologies which can be found in the CMTs category are CollegaMENTI for Silver Age and CuraMI.Tech. UNMTs, instead, include for example: Amyko and Canary Care.

The second largest category of ETs is that of SARs (see Table 3), and, among them, the ones which can be classified as Service-Type Robots (e.g. Alfred and Care-O-Bot 3), are more than double the ones which are classified as Companion-Type Robots (e.g. Paro and Pepper).

VRTs represent a small fraction of all ETs (see Table 3), as this category includes only eight technologies, e.g. Granny Vision and Kaleido.

**Table 3:** Types of ETs

Types of ETs^a^		N. of ETs	% of ETs
Monitoring		182	82
1	CMTs	38	17
2	UNMTs	144	65
SARs		34	15
1	Companion-type	7	3
2	Service-type	28	12
VRTs		8	3

^a^ Two technologies (Alfred and LISA habitec) can be classified both as UNMTs and as service-type SARs. One SAR (Samsung Bot Care) can be classified both as a service-type SAR and as a companion-type SAR

#### Functions of ETs

Most technologies present more than one use, since several ETs bring together in a single device a combination features which make the technology useful on several fronts: these devices have therefore been included in the count for multiple functions (for instance, most wearable devices have both GPS tracking and fall detection functions). ETs’ most common functions are listed in Table 4.

**Table 4:** Functions

Functions	N. of ETs	% of ETs
Monitoring	133	60
Emergency detection &/or calls	76	34
Fall detection	72	32
Assistance in managing healthcare (e.g. giving reminders)	51	23
GPS Tracking	38	17
Social connectivity & communication (with family, caregivers, friends, etc.)	34	15
Predictive analytics (memorizing habits & prescribing insights)	26	11
Companionship & interaction	25	11
Cognitive training, stimulation & rehabilitation	24	11
Entertainment	20	9
Collecting healthcare data	19	8
Psycho-social support & coaching	15	7
Assistance in practical activities (e.g. delivering objects)	13	6
Physical rehabilitation & exercise	11	5
Planning	7	3
Robotic Manipulation^b^	4	2
Helping and alerting nurses and caregivers	2	1
Communication with healthcare professional	1	< 1

^b^ This expression refers to practical and manual activities such as picking up and delivering objects to patients, opening jars, grasping different utensils, and performing even quite difficult tasks such as preparing a meal

A large amount of technologies have monitoring functions, which we distinguish between what we referred to as “passive monitoring” and “active monitoring”, in relation to the extent the elderly person is involved in the monitoring activity. Passive monitoring refers to technologies which can monitor health and wellbeing automatically, so they do not require any action from the elders (e.g., domotic systems or environment sensors). Active monitoring, instead, implies that the elder is actively involved in managing the technology and monitoring his health (i.e., through wearables), therefore some digital literacy is necessary.

The second most common functions are emergency detection and/or emergency calls, provided for example by HomeGuardian and Kompai Robot. In addition, a fraction of identified devices can specifically detect falls (e.g., HOBBIT and Kanega).

Some technologies give support and assistance in managing healthcare: ElliQ and Romeo, for instance, can send reminders and help patients to comply with medical prescriptions, in this way facilitating the caregivers’ work.

Other important functions are improvement of social connectivity and communication. SARs, such as Care-O-Bot 3 and ElliQ, can contribute to satisfying older adults’ need for companionship, interaction and/or entertainment.

Several devices provide cognitive training, facilitate stimulation and rehabilitation, or give psycho-social support and coaching. Others can help in physical rehabilitation and exercise.

A fraction of technologies can assist in practical and manual activities such as picking up and delivering objects to patients. Some ETs can also open jars, grasp different utensils, and perform even quite difficult tasks such as preparing a meal. These latter, more complicated functions are referred to as robotic manipulation and are performed especially by Alfred and Tiago Robot, which are equipped with proper robotic arms.

Some devices can collect healthcare data, memorize habits and offer insights, giving also predicting analytics. Mymedbook, Sara and Visavis, in particular, give assistance in planning.

Finally, another emerging feature is GPS tracking, which is present in a high number of technologies.

#### Target of ETs

With regard to the target, we did not observe significant differences in the gender of the target population, as all technologies are equally suitable for males and females. However, we did notice a differentiation of the target in relation to the degree of older adults’ autonomy (see Table 5).

**Table 5:** Target

Target	N. of ETs	% of ETs
All seniors: Autonomous seniors; Semi-autonomous seniors; Seniors with MCI and/or Dementia and/or Alzheimer’s disease	175	79
Autonomous seniors Semi-autonomous seniors	24	11
Semi-autonomous seniors Seniors with MCI and/or Dementia and/or Alzheimer’s disease	5	2
Seniors with MCI and/or Dementia and/or Alzheimer’s disease	14	6
Autonomous seniors	2	1
Semi-autonomous seniors	2	1

And, while some websites clearly state the target for which the ETs are designed, in other cases this was inferred from the technologies’ characteristics.

A major portion of technologies are developed for all seniors: autonomous, semi-autonomous, and seniors with Mild Cognitive Impairment (MCI) and/or Dementia and/or Alzheimer’s disease.

The rest of the technologies are more specifically targeted to certain subgroups of seniors: some are intended for autonomous and semi-autonomous seniors (e.g., Alfred and MAGIC-GLASS), while others are intended for semi-autonomous seniors and seniors with MCI and/or Dementia and/or Alzheimer’s disease (e.g., CareMat and Ti-Seguo).

Some ETs are designed exclusively for one specific group of seniors: for seniors with MCI and/or Dementia and/or Alzheimer’s disease only (e.g., AngelSense and SensorNet), for autonomous seniors only (MoveCare and NoonCare), or for semi-autonomous seniors only (Vitalerter and Robear).

#### Time of development and its relation to COVID-19 pandemic

ETs for elderly care were mainly developed before the COVID-19 pandemic (Table 6). Nevertheless, in proportion, it is noteworthy that 14% of all ETs have been developed during the pandemic. Of these, the majority are UNMTs: in particular, 10 technologies are wearable devices, while 11 UNMTs are Ambient Intelligence devices. Finally, one technology (Aph-Alarm) consists of both wearable sensors and environmental sensors. Of the remaining technologies developed during the pandemic, 4 are CMTs, 4 are SARs and, finally, only 1 is VRT.

**Table 6:** Time of development

Time of development	N. of ETs	% of ETs
Before COVID-19 pandemic	191	86
During COVID-19 pandemic	31	14

#### Additional information on ETs

Some additional information was collected about the location where the identified ETs were first developed and the type of funding they received.

Considering the locations (see Table 7), we may notice that a large percentage of technologies come from two countries: Italy (e.g. CloudIA, E.CA.RE, Kibi Wear, Robot R1, WiMHome) and the USA (e.g. Alexa Together, AngelSense, LUNA lights, Pria, QuietCare). As mentioned above, such a high percentage of Italian technologies may be certainly explained on the basis that the keywords search was performed both in English and in Italian, so as to purposedly identify the largest possible number of technologies developed in Italy. A further analysis of selected Italian ETs showed that a good share of them were developed in Lombardy.

Other countries which represent locations for ETs development are: the UK, Switzerland, France and Israel, Germany, Finland, Australia, Czech Republic, Spain, Japan, China, Belgium, India, Denmark and South Korea, Austria, Ireland, the Netherlands, Norway, Singapore, Tunisia, Turkey, United Arab Emirates.

Finally, some technologies were the result of multi-national projects sponsored by the European Union (EU).

**Table 7:** Locations

Locations	N. of ETs		% of ETs		
USA	76		34		
ITALY	Total	Lombardy	Total		Lombardy
	41	13	18		6
UK	34		15		
MULTI-NATIONAL (EU PROJECTS)	13		6		
SWITZERLAND	9		4		
FRANCE	5		2		
ISRAEL	5		2		
AUSTRALIA	4		2		
GERMANY	4		2		
FINLAND	4		2		
CZECH REPUBLIC	3		1		
JAPAN	3		1		
SPAIN	3		1		
BELGIUM	2		1		
CHINA	2		1		
DENMARK	2		1		
INDIA	2		1		
SOUTH KOREA	2		1		
AUSTRIA	1		< 1		
IRELAND	1		< 1		
THE NETHERLANDS	1		< 1		
NORWAY	1		< 1		
SINGAPORE	1		< 1		
TUNISIA	1		< 1		
TURKEY	1		< 1		
UNITED ARAB EMIRATES	1		< 1		

Given the focus on Italy, deriving from the rationale of the project in which this work is framed, it is interesting to note which technologies have been developed on the Italian soil. Our findings show that in Italy (and in Lombardy, in particular) mostly UNMTs were developed, of which 15 are Ambient Intelligence technologies, 10 are wearable devices, and one technology has a twofold function, presenting both wearable and Ambient Intelligence components. CMTs represent the second most numerous ETs class (8 devices). Concerning the other two categories, we identified only one VRT and two SARs (service type), of which one is combined with components of Ambient Intelligence.

As to the development period, the growing interest in ETs for the elderly care is shown by the fact that 30% of the 41 technologies developed in Italy (N = 12) were designed and marketed during the pandemic time. All of these 12 ETs are monitoring technologies, of which 8 belong to UNMTs.

Regarding sources of funding (see Table 8), our analysis shows that private companies are responsible for funding a significant share of ETs (e.g., Amyko and Romeo), plus a few technologies which were developed by a partnership of private companies (GAP and BrainMEE). A second smaller group of ETs were the result of research projects launched in response to national and international calls, funded by universities, profit and non-profit partners, etc. (e.g., CollegaMENTI and MARIO). The remaining technologies are roughly half resulting from projects of research institutes and/or universities (e.g. Care-O-Bot 3 and Robot R1) and half resulting projects of non-profit associations and/or universities and/or research institutes and/or private companies (e.g., CuraMi.Tech and Isidora).

**Table 8:** Funding

Funding	N. of ETs	% of ETs
Private Companies	187	84
Research projects in response to national and international calls for proposals with universities, profit and non-profit partners, etc.	21	9
Projects of non-profit associations and/or universities and/or research institutes and/or private companies	6	3
Projects of research institutes and/or universities	6	3
Partnership of private companies	2	1

Finally, as to the developmental stage, we found that a large proportion of ETs are already on the market; the remaining are still in the project phase, except for one technology, which is out of market (Pepper the robot).

### Ethics-related analysis

#### Theoretical background

While doing data extraction and synthesis, we realised that most websites referred to what has been labelled elsewhere as “ethically-related contents” [16, p. 5]. With this expression we refer to contents dealing with ethical issues as interpreted within well-known ethical approaches (e.g., principlism, care ethics). Despite not applying a unique pre-conceived ethical framework for the qualitative investigation of the website contents, the top-down approach that the work originally set out was relational care ethics, which is grounded on the conviction that there is moral significance in the fundamental elements of (care) relationships and dependencies in human life. This approach was operationalised in a dignity enhancing model of care [24] where not only dignity but also vulnerability play a central role [25].

However, while analysing the results of our research, we also adopted a bottom-up approach which brought forth some considerations that build on different ethical approaches. Firstly, principlism, which represents the major approach of contemporary Anglo-Saxon biomedical ethics, and it is based on four prima facie moral principles (autonomy, non-maleficence, beneficence, and justice) [26]. Secondly, some issues referred to public health ethics, an approach which focuses on the problem of health, considered not only as an individual condition, but also as a complex phenomenon, concerning the entire population, and is aimed at providing concrete moral guidance to pursue the health of the public as the ultimate end [27]. Finally, a reflection was also made on the link between technology development and the CoviD-19 pandemic.

#### Ethical contents

While presenting ethically-related results, we are going to adopt the framing of the technologies’ developers; accordingly, potential critical aspects will not be covered in the following paragraph, as they are hardly ever mentioned in the ETs’ websites.

However, in the discussion section, all the positive values which emerge from the analysis of the ETs on the market will be further discussed by reflecting on the related ethical implications and possible criticalities or concerns. It should be also pointed out that most websites simply list the technology’s benefits in very broad terms, without referring to academic literature or analysing them in depth.

The most recurring positive aspects mentioned when addressing the impact of ETs are: safety; independence and active aging; connectedness; empowerment and dignity; engagement and entertainment; cost and efficiency.

### Safety

Feeling safer when being alone is a need that is keenly felt by most people when getting older. Therefore, the first and most common expected result when introducing technology in the care of an older person is greater safety, both from a physical and a psychological point of view [28]. A good portion of ETs aims at increasing the elder’s safety during everyday life activities, especially those wearables and technologies with monitoring functions, fall and emergency detection functions, or GPS tracking functions (i.e. SEREMY, Tahoma 2.0, Ti-Seguo, etc.). Other ETs, such as cognitive and emotional assistive systems or smart health monitoring systems, further contribute to the sense of relief and greater security (i.e. care.coach, HealthyTogether, etc.).

ETs with monitoring functions increase safety because they enable constant monitoring of the vital parameters and health condition of the elderly, whether healthy or ill. In this way, these devices can reduce older adults’ physical vulnerability, related to “bodily deterioration”, i.e. “non-pathological and pathological physical/physiological decline” [25, p. 11].

Knowing that their health condition is being monitored, the elderly can feel calmer and safer: they know their caregivers are constantly updated on their status and ready to intervene in case of emergency. Accordingly, ETs reinforce a sense of security, instilling peace of mind and reducing the stress associated with the perception of an instable psycho-physical condition. In this sense, ETs can also tame the psychological vulnerability of the elderly, which is related to emotional factors (e.g., anxiety and fear of being unsafe, especially in the absence of caregivers) and to experiential components, for instance, feeling a ‘decaying body’ [25, p. 11].

In line with these considerations about safety and physical and psychological well-being, most of the technologies developed during the COVID-19 health emergency are UNMTs. The necessary imposition of containment measures, aimed at avoiding virus transmission, has in fact made health monitoring and verification more complex, thus potentially putting elderly people at risk. The design and development of UNMTs has been a useful aid to partially overcome these problems, which affected both the independent (or partially independent) elderly living in private homes and the institutionalised elderly. In fact, UNMTs make it possible to check health parameters remotely, avoiding the risk of infection for both elderly people and caregivers and/or family members. Nevertheless, the development of CMTs and SARs during the pandemic has also been a useful resource, from other points of view, and it should be seen in this same light.

By increasing the physical and psychological well-being of the elderly and their protection, ETs also have a potential positive impact on older adults’ Quality of Life. Therefore, in the light of the principles of biomedical ethics [26], undoubtedly the use of these tools promotes the principle of *beneficence.* A positive impact can be observed also with regard to *non maleficence*: by constantly monitoring older adults, ETs allow prevention and emergency response, thus enhancing safety and protection of older adults, especially of the frailest ones [15].

### Independence and active aging

Despite the physical and psychological limitations which might accompany old age, elderly people do not want to lose their independence and wish to live their lives without being overly reliant upon others. For this reason, many ETs seek to enable seniors to live in their houses independently, by performing all those tasks for which they would require the help of a caregiver or a family member [19, 29] SARs, such as Mylo, Hector, HOBBIT, Romeo etc., are important tools for assisting and providing support to elders both from a practical and from a psychological standpoint. All the ETs with prevention functions (e.g., Zibrio, CarePredict Tempo) also preserve older people’s independence as they avoid the worsening and further decrease of their residual capacities. Wearables, instead, like AngelSense and In Touch, give seniors the freedom to move around and even leave their houses, while being monitored for safety. This increases elders’ independence and promotes their active and healthy aging, while also relieving caregivers and family members from worry and the need to be constantly present.

The boost of independence, in the context of principlism, can be regarded as promotion of the principle of *autonomy*, intended as older adults’ self-determination and capacity for autonomous action. Indeed, ETs allow the elderly to keep carrying out daily tasks and engaging in their favourite activities (e.g., doing physical activity and/or meeting friends and/or cooking) in partial independence. By allowing older adults to maintain a certain degree of autonomy, ETs can also have a positive impact on the principle of *beneficence* [26]: performing more activities on their own, supported by ETs but without the intervention of a caregiver, helps older adults retain certain abilities and prevents their early deterioration. In this way, the use of ETs reduces seniors’ perception of losing control and being increasingly dependent on others, a feeling which is typically associated with the relational vulnerability of the elderly [25], as emphasised by relational care ethics approaches. ETs can therefore be said to tame this kind of vulnerability by allowing older adults to feel more self-reliant.

Even during the pandemic, ETs (especially CMTs, UNMTs and SARs) have made an important contribution. Indeed, they have enabled the elderly to partially compensate for the absence of care at home or for the lack of assistance from family members and/or caregivers, caused by the measures to contain the spread of SARS-CoV-2.

### Connectedness

Considering older adults’ need for relationships, companionship and interaction, many ETs have been developed to enhance their connections with family, friends, as well as caregivers and healthcare professionals. ETs such as VitalBand and Care-O-Bot 3 make it a lot easier for seniors to get in touch with their loved ones, both through messages and videocalls. This reduces older adults’ isolation and allows them to remotely communicate more often with family members. Many technologies and platforms, like MoveCare and CollegaMENTI for Silver Age, are also designed to create virtual networks between seniors and build a community, in order to facilitate their participation in social activities.

The impact that ETs have on connectedness, from an ethical point of view, and specifically in the context of relational care ethics approaches, translates into implications for *relational autonomy*, as personal self-determination and freedom can be regarded as dynamic phenomena that can only flourish in the context of human relationships [30]. In fact, by facilitating communication with caregivers, relatives and friends, ETs enable older adults to be more actively involved in their circle of relationships. The creation of a stronger network of relationships, achieved through the use of ETs, also reduces the elderly’s sense of loneliness. This may prompt older adults feeling less vulnerable from the psychological standpoint [25], as negative feelings connected to isolation and loneliness are reduced. Relational vulnerability may also appear tamed: older adults may feel vulnerable because they lack “adequate family support” and/or because they live alone and feel lonely [25, p. 11]: with ETs, social relations can be easily cultivated even at a distance, fulfilling the elderly’s need for company and support.

The pandemic experience, in this regard, brought to the light these advantages of ETs. The various types of ETs, in fact, enabled the elderly to fulfil their need for sociability, to maintain relationships with family and friends, and to meet their need to cultivate social activities, even during the period of physical distancing.

### Empowerment and dignity

By implementing the use of ETs which are constantly monitoring health parameters and behaviours, or providing reminders and suggestions, older adults are inevitably made more aware of their physical and psychological status. Using these technologies empowers them to take charge of their health condition and pushes them to learn how to better take care of themselves autonomously. This has a great impact on elders’ dignity because, assisted by technology, they feel that they can manage their life even in their older age. In this sense, wearables, smart home systems and SARs can be extremely useful for older adults’ empowerment [16], making them responsible for their own well-being.

The promotion of older adults’ dignity and the recognition of their decision-making capacity and responsibility are one of the main pillars of relational care ethics, which is aimed at considering and treating the elderly as a person [24]. Therefore, the use of ETs has significant ethical implications also from this perspective. The use of ETs in fact allows older people to feel that they are still autonomous subjects, whose choices and actions deserve respect. In this way their dignity is also recognised and enhanced. For similar reasons to those considered for dignity enhancement, also the principle of autonomy can be fostered by using these devices: the personal involvement in self-care increases older adults’ perception of self-determination and gives the elderly a sense of leadership in managing their own health. By promoting empowerment, ETs can also tame moral vulnerability, strengthening the elderly’s self-confidence and a sense of their own dignity.

### Cost and efficiency

As the costs of healthcare services are ever rising, the introduction of technology in the care of the elderly needs to be considered also in terms of cost-effectiveness. Cost reduction, indeed, is one of the main interests among patients, families, and healthcare managers. ETs could be beneficial as they have the potential to create significant cost savings. Many home-based monitoring systems (e.g., SmartCare, Salute a casa, Independa Health Hub) can be used for telemedicine and accessed by physicians and nurses, sparing older adults the need to reach hospitals and doctors’ offices for visits, saving time and travel costs. The dissemination of technology can make it possible to guarantee health services even for those living in rural areas. Furthermore, technologies with fall prevention functions (e.g., Zibrio) avoid hospitalisation and the related costs. Finally, the introduction of SARs partially caters to the rising demand for healthcare workers.

Considerations in terms of cost and efficiency raise questions of public health ethics. This approach considers older adults’ vulnerability as a multi-layered condition that results also from socio-economic factors. Undoubtedly, reflecting on the positive outcomes outlined above, the implementation of ETs can have a positive impact also from this point of view, taming the socio-economic vulnerabilities of the elderly.

The pandemic context emphasized the benefits of ETs also in relation to the socio-economic dimension: technological devices allowed the remote management of some care practices, thus proving their cost-efficiency compared to traditional visits and monitoring in hospital. As to the social component, ETs made it possible to prevent infection.

## Discussion

The in-depth mapping of ETs just described in its main findings provides a complete overview of the current use of technology in elderly care and gives way to some reflections and ethical considerations. By analysing all the information gathered and focusing particularly on the specific functions of the identified ETs, the authors have noticed that, while a great variety of benefits are listed as expected results of technology use, a critical counterpart also exists, and some often-overlooked problematic aspects and potential threats emerge. In this sense, it is impossible not to notice the ambivalence of the use of such technologies in aged care. Therefore, in line with current trends in academic debate, some ethical criticalities may be also pointed out.

### Safety and liability

As previously stated, safety is one of the positive aspects promoted by the use of ETs. However, the fact that some technologies could also threaten the security of older adults as technical devices could have malfunctions and cause accidents should not be underestimated. In this way, paradoxically, ETs can exacerbate physical and psychological vulnerabilities, potentially causing physical harm to the elderly and subjecting them to additional stress [25]. This raises important questions related to moral responsibility and legal liability [16]. In the case of an injury or other kinds of damage, it is difficult to determine who should be held responsible (programmers, engineers, caregivers or others).

However, such risks to the safety of the elderly could be addressed in a very practical way, through further technical improvements. One possible solution, should ETs malfunction, could be the provision of an emergency button that is always on, or an automatic warning system that sends messages to caregivers. Another possible countermeasure is the provision of a training service for the elderly, if they are still capable and autonomous, or for caregivers and/or family members, to make sure that users know how to appropriately use technology. Whether the elderly are capable and autonomous or not, the main prevention is, however, the presence of (or the possibility of contacting) a mediator in the use of ETs (caregiver and/or family member and/or health professional).

### Privacy and surveillance

All the ETs’ functionalities which allow monitoring and the increased degree of safety often pose questions in terms of privacy [31]. The use of monitoring technologies and SARs represents a significant threat to older adults’ privacy, both intended as informational privacy and physical privacy [28, 32, 33].

Informational privacy encompasses the collection, storage, use, maintenance, dissemination/disclosure, and disposition of personal information (including data related to a person’s vital parameters, physical functions, behavioural patterns and locations) [34]. The violation of this kind of privacy can result from third party’s access, control, and abuse of the collected data. Therefore, practical enforcement of effective data protection measures is crucial and it would be preferable if seniors had personal control over the data collected [35].

Physical privacy, instead, relates to the invasiveness, intrusiveness and obtrusiveness of ETs [32]. The use of video cameras, for instance, can cause the so called *Big-Brother Syndrome* [2], which means that older people may have the perception of being constantly observed by the monitoring systems and feel bewildered by the presence of numerous devices, which invade their personal space [33].

Older adults are often willing to accept some privacy infringements in favour of the safety and other benefits provided [3], but despite this trade-off, they still require technology to be reliable and trustworthy [36]. Sometimes the promise of safety could lead elders to accept technologies before actually being ready and making compromises which do not benefit their wellbeing [33].

The negative consequences of ETs on privacy result in the exacerbation of the vulnerability of the elderly in many respects. First of all, these devices worsen the loss of control, because older adults can feel themselves “illegitimately surveilled by others” [25, p. 12]. In this sense, the use of ETs may threaten older adults’ autonomy [26], in the sense of self-determination and freedom from external interference. To the extent that a person feels (negatively) observed, he/she perceives the gaze of others as an illegitimate interference in his or her life.

Secondly, ETs exacerbate relational vulnerability: the elderly, perceiving the constant monitoring by caregivers, may feel even more dependent on others [25, p. 11]. The worsening of psychological vulnerability is therefore also inevitable, due to negative emotions related to the feeling of continuous surveillance. The principle of beneficence is thus also partly violated, as ETs impair psychological and emotional well-being.

All these critical aspects undoubtedly have an impact on the moral vulnerability of the elderly, potentially damaging their dignity. The pervasive use of monitoring technologies might make the elderly feel that they are being treated as objects, causing the phenomenon of infantilisation [25, p.11]. Finally, insofar as ETs enable a kind of bio-political control of the life of the elderly population, the use of such technologies may also be relevant in relation to the dimension of political vulnerability [25, p. 12], which is related to the (negative) treatment of aged people as a social group.

To avoid (or contain) the aforementioned risks, a first measure is undoubtedly an adequate communication with the elderly person, in order to provide information to the extent appropriate to his or her ability to understand. In so doing, older adults, if capable and autonomous, can take a more informed decision to use the ETs or not.

Regarding Big-Brother Syndrome, one possible solution to reduce privacy threats is the use of sensor systems which do not include cameras or at least do not collect high-definition images in the private rooms of the house (such as the bathroom). The elderly person should also be given a choice as to who can have access to such information (data, images, and/or sounds).

### Autonomy and consent

The ethical theme of privacy is strictly connected to that of autonomy. The use of ETs has the potential of promoting older people’s independence and autonomy. However, some measures must always be implemented to ensure that new assistive technologies do not become instruments of surveillance and control, diminishing the autonomy and the independence they intend to promote [37], as we have seen above.

As stated in the previous paragraph, one essential measure is the informed consent procedure [33, 34, 38, 39]. Informed consent cannot be reduced to the mere acceptance of the product’s terms of service [40]. Older adults must be made aware of each function of the technologies they are using, and they must be informed in a way that is compatible with their cognitive abilities, ensuring that they understand the technology’s expected benefits and risks [33, 40, 41].

### Connectedness and isolation

As seen above, a great advantage of ETs is that they allow older adults to be more connected to family, friends and healthcare professionals.

Nevertheless, it must be considered that this connection always happens through a device and cannot be compared to in-person visits. Although wearable devices represent a promising tool for promoting the health interests of isolated older adults, technology cannot replace social connections and the lack of human touch is likely to be distressing for older adults [42, 43].

In this sense, paradoxically, ETs can exacerbate isolation and loneliness, jeopardising social connectedness and other values, such as love, empathy and human touch [2, 32, 33, 44]. In fact, relying on the existence of monitoring technologies and robots, home visits and visits in nursing homes might decrease, leaving the elderly to feel neglected or even abandoned. As a result, on a societal level, ETs’ abuse or misuse can cause dehumanization of care, due to the progressive replacement of human interaction by human-technology interaction.

From this point of view, ETs can negatively impact not only psychological and relational vulnerabilities, exacerbating the sense of abandonment in the elderly, but also, in general, their socio-cultural vulnerability, because they might feel “excluded from social life and/or isolated” [25, p. 12].

Therefore, it is of pivotal importance that the use of technology does not become the only means for elderly people to communicate and be connected to family, friends and healthcare professionals. Older adults should be reassured about the fact that ETs can be introduced as a useful tool to maintain and cultivate relationships, but they will not replace in-person networking and care.

### Empowerment and dignity

A correct and conscious use of ETs enables older adults’ empowerment and promotes their dignity. In contrast, the abuse of technology can cause depersonalization and infantilization, exacerbating moral vulnerability: older adults, perceiving themselves as constantly monitored by others, may think they are being treated as objects or infants [25]. Another negative aspect derives from the fact that some devices are also held responsible for stigmatization in public: the elderly might be ashamed to use such technologies, because they do not want to be stigmatized, i.e. considered incapable of taking care of themselves.

Making older adults comfortable with the presence of technology in their lives has become one of the main goals of ET developers, who aim at designing devices which are always less intrusive and noticeable. Hopefully, this will prevent stigmatization and protect elders’ dignity.

### Cost and efficiency

While in the long-term ETs could lead to a significant reduction of healthcare costs and an improvement in the access to health services [45], at present these technologies (wearables, home automation systems and especially robots) are often very expensive and cannot be accessed by most families; for instance PARO robot costs around 6.000 €. This creates a serious problem of affordability and social justice [33, 46]. Accordingly, ETs can exacerbate elderly’s economic vulnerability [25]. In light of the principlist approach, ETs may have a negative impact in relation to the principle of *justice* [26], because the unequal distribution of ETs could exacerbate sociocultural inequalities.

Evidently, the other unavoidable issue to be considered is fair access [33], which is closely linked to the problem of a possible worsening of the so-called “digital divide” [3], with regard to social justice, and the socio-cultural vulnerability of older adults [25].

Affordability and access issues will probably be addressed over time: at present, many ETs are very expensive because they are not produced and marketed on a large scale, and some are still in the project or prototype phase; however, as technology becomes a more widespread tool for healthcare management, ETs costs will hopefully lower and a greater number of older people will have the possibility to purchase them and use them in their daily lives.

### Alienation and deception

A final critical issue which must be considered is the risk for older adults of being deceived by technologies, which can create a feeling of alienation, disengage elders from reality and exacerbate psychological vulnerability. Focusing attention on humanoid robots, like Romeo, older adults can have conscious and unconscious delusions about the machine’s real intellectual and emotional capabilities [28, 44]. Older adults might feel sad or frustrated, because their emotions are not mirrored and/or rewarded: potential negative effects of this phenomenon are objectification and infantilisation [28, 44]. Users might perceive robots as “toys” and, as a consequence, they might think caregivers treat them like children. In this sense, ETs may also worsen dimensions of moral vulnerability [25].

In order to avoid these risks, caregivers and family members should closely monitor the perception which older adults have of robots or other ETs, especially when they are initially introduced to the new technology. Observing how the technology is perceived by elders is important to pick up any early sign of deception or alienation and, in that case, intervene to raise awareness about the actual nature and role of the ET in their life, thus making sure that the relationship with technology stays conscious and healthy.

## Strengths and Limitations

This research is an important contribution to the debate on technologies employed in aged care, as it analyses a large number of ETs, specifically targeted to older adults, and focuses on recently developed devices, in order to also intercept possible influences of the exceptional pandemic situation. In addition to significantly enriching the international academic debate on the role of ETs in aged care, this research contributes to the broader project *ElderTech – Emerging Technologies and Vulnerabilities in Aged Care*, which aims to comprehensively investigate and delineate the concept of vulnerability when referred to the ageing population and to study the impact of ETs on older adults’ vulnerability, in the context of care, broadly understood.

Another notable positive feature of this study is the ethical review proposed, which covers both promoted values and critical aspects, highlighting the *inherent ambivalence* of technological development.

The main limitation of this work is that the sample of technologies is only partially representative because the web search was performed using only English and Italian keywords. This may have potentially introduced a bias in the ETs collection, with a prevalence of Italian technologies retrieved from the web search. This, however, allowed us to gain robust evidence on this national reality.

## Conclusions

This work was purposed to provide an in-depth and updated overview of the technologies currently employed in elderly care. This objective was achieved firstly, by mapping and classifying the ETs currently available on the market and, secondly, by assessing the impact of such ETs on elderly care, exploring (ethical) values promoted, as well as potential ethical threats.

This research makes an important contribution to elderly care by proposing potential meaningful content for all stakeholders involved in the field: technology developers can draw decisive information on the technologies which are preferred by consumers and reflect on how they can improve ETs to solve some of the issues emerging from the ethical analysis; elderly people will benefit if this contribution stimulates the production of ETs that are more suitable to their needs and preferences, protecting their dignity and autonomy; policy makers can obtain information on the deployment of ETs, on their functions, as well as on their advantages and flaws, which are important aspects that policies should address; professionals working in the assistance and healthcare sectors (managers of residential centres, caregivers, health workers), can gain insights about the possible critical aspects of the use of ETs and can subsequently be more aware in their daily practice.

Finally, as far as the academic sector is concerned, the research adopts an innovative perspective, interweaving market analysis with theoretical reflection and combining the ethics of ETs with considerations from the ethics of care, principlism, and public health ethics.

The research on market trends shows the clear interest in the development of ETs for the care of the elderly, as shown also by the data regarding the pandemic period.

Undoubtedly many are the benefits of ETs, emphasised by companies, research centres, and academics as well. However, attention should also be paid to potential threats, critical aspects and ethical challenges, as widely reported in academic literature, while less theorised by stakeholders involved in the design, development, and dissemination of technologies themselves.

## Electronic supplementary material

Below is the link to the electronic supplementary material.

Supplementary Material 1

## Data Availability

All data generated or analysed during this study are included in this published article [and its supplementary information file].

## List of abbreviations

CMTs: Conventional Monitoring Techniques
ETs: Emerging Technologies
EU: European Union
MCI: Mild Cognitive Impairment
SARs: Socially Assistive Robots
UNMTs: Unconventional Monitoring Techniques
VRTs: Virtual Reality Technologies
