# Supplementary material for: Mapping emerging technologies in aged care: results from an in-depth online research
Source: BMC Health Serv Res. 2023 May 23;23:528. doi: 10.1186/s12913-023-09513-5 (PMC10204691; doi:10.1186/s12913-023-09513-5)
Supplement: Supplementary file 1 — Supplementary Material 1 [file 12913_2023_9513_MOESM1_ESM.docx]

**Additional File 1**

|  | **Technology** | **Type of ETs** | **Website where the information was retreived** |
| --- | --- | --- | --- |
| 1 | ACT Assess and Connect | CMT | https://agecaretechnologies.org/ |
| 2 | Addison.Care | UNMT – Ambient Intelligence | https://addison.care/ |
| 3 | ADT Home Security | UNMT – Ambient Intelligence | https://www.adt.com/resources/safety-tips-for-the-elderly |
| 4 | Alexa + Echo / CareHub | UNMT – Ambient Intelligence | https://www.businessinsider.com/guides/tech/how-to-use-alexa-care-hub?r=US&IR=T |
| 5 | Alexa Together | UNMT – Ambient Intelligence | https://www.amazon.com/Alexa-Together/b?ie=UTF8&node=21390531011 |
| 6 | Alfred | UNMT – Ambient Intelligence + SAR Service Type | https://alfred.eu/ |
| 7 | Amyko | UNMT – Wearable | https://www.amazon.it/Amyko-AMKNR01-Bracciale-Emergenza-Nero/dp/B01A8QZ730 |
| 8 | Ancelia | UNMT – Ambient Intelligence | https://teiacare.com/ancelia/ |
| 9 | AngelSense | UNMT – Wearable | https://www.angelsense.com/gps-tracker-for-elderly/ |
| 10 | Aph-Alarm | UNMT – Wearable & Ambient Intelligence | https://www.aal-europe.eu/projects/aph-alarm/ |
| 11 | AskMarvee | UNMT – Ambient Intelligence | https://voiceincanada.ca/alexa-and-aging-with-heidi-culbertson-from-marvee/ |
| 12 | Bluetooth Low Energy Bracelet | UNMT – Wearable | https://rfid.it/il-bluetooth-che-aiuta-gli-anziani/ |
| 13 | Bodyport | UNMT – Ambient Intelligence | www.bodyport.com |
| 14 | BrainMEE | CMT | https://brainmee.com/en/our-offer/ |
| 15 | Canary Care | UNMT – Ambient Intelligence | https://www.canarycare.co.uk/ |
| 16 | Care@Home | UNMT – Wearable & Ambient Intelligence | https://www.essencesmartcare.com/ |
| 17 | Care-O-Bot 3 | SAR Service Type | https://www.care-o-bot.de/en/care-o-bot-4.html |
| 18 | care.coach | SAR Service Type | https://www.care.coach/ |
| 19 | Care Alert | UNMT – Wearable | https://secom-caretech.co.uk/care-alert/ |
| 20 | CareBand | UNMT – Wearable | https://www.carebandremembers.com/ |
| 21 | CareGo | UNMT – Wearable | https://secom-caretech.co.uk/care-go/ |
| 22 | Care Hub | UNMT – Wearable | https://secom-caretech.co.uk/care-hub/ |
| 23 | Care Hub Plus | UNMT – Wearable | https://secom-caretech.co.uk/care-hub/ |
| 24 | CareIP | UNMT – Ambient Intelligence | https://www.mckinsey.com/about-us/new-at-mckinsey-blog/how-an-award-winning-design-is-helping-older-adults-live-safely |
| 25 | Careline Digital Alarm | CMT | https://www.careline.co.uk/ |
| 26 | Careline Fall Alarm | CMT | https://www.careline.co.uk/ |
| 27 | Careline Pendant Alarm | CMT | https://www.careline.co.uk/ |
| 28 | Careline SOS GPS Alarm | CMT | https://www.careline.co.uk/ |
| 29 | CareMat | UNMT – Ambient Intelligence | https://osatech.ch/telesoccorso/chiamata-infermieri-tappeti-caremat.html |
| 30 | CarePredict Tempo | UNMT – Wearable | https://www.carepredict.com/press-releases/new-research-shows-carepredict-improved-health-outcomes-and-staff-engagement-in-senior-living-facilities/ |
| 31 | CareVoice x Tempo | UNMT – Wearable | https://www.carepredict.com/press-releases/carepredict-introduces-carevoice-at-ces-2022-your-voice-on-their-wrist/ |
| 32 | CARU | UNMT – Ambient Intelligence | https://www.caru-care.com/ |
| 33 | CASPAR.AI | UNMT – Ambient Intelligence | https://caspar.ai/ |
| 34 | Cherry Home | UNMT – Ambient Intelligence | https://get.cherryhome.ai/care/ |
| 35 | Closer | UNMT – Ambient Intelligence | https://www.tigahealth.com/p/closer-elderly-smart-home/ |
| 36 | CloudIA | UNMT – Wearable & Ambient Intelligence | https://www.ergonomicsdesignlab.com/cloudia |
| 37 | CollegaMENTI for Silver Age | CMT | https://www.collega-menti.it/ |
| 38 | Comarch con te | UNMT – Wearable | https://www.comarch.it/healthcare/prodotti/telemonitoraggio/remote-care-services/life-wristband/ |
| 39 | CUraMI.Tech | CMT | https://curamitech.wordpress.com/ |
| 40 | Cutii | SAR Service Type | https://www.cutii.io/en/ |
| 41 | Dfree | UNMT – Wearable | https://www.dfreeus.biz/ |
| 42 | Digital Fall Alarm | CMT | https://taking.care/pages/fall-alarm-for-elderly |
| 43 | Digital Health Coach | CMT | https://www.ageingtech.it/telemedicina-digital-health-coach/ |
| 44 | Digital Personal Alarm | CMT | https://taking.care/pages/no-landline-digital-personal-alarm |
| 45 | DOMEO | SAR Service Type | http://www.aal-europe.eu/projects/domeo/ |
| 46 | Domotic Kit | UNMT – Ambient Intelligence | https://www.altoadigeinnovazione.it/tecnologia-terza-eta/ |
| 47 | Dr Versa | SAR Service Type | https://becominghuman.ai/adding-a-new-dimension-to-elderly-care-and-healthcare-with-voice-technology-128c73e1e28a |
| 48 | E.CA.RE | UNMT – Wearable | https://ecareproject.eu/ |
| 49 | Echo (Elderly Care Home Observing) System | UNMT – Ambient Intelligence | http://www.echocare-tech.com/ |
| 50 | Electronic Life Record - ELR | CMT + UNMT – Wearable | https://lifespark.com/ |
| 51 | ElliQ | SAR Service Type | https://elliq.com/ |
| 52 | Elma Smart Detection | UNMT – Ambient Intelligence | https://www.maricare.com/en/how-it-works/elma-smart-detection |
| 53 | Elro | SAR Service Type | https://www.elro.ai/ |
| 54 | eLsa™ Activity Sensing | UNMT – Ambient Intelligence | https://www.maricare.com/en/how-it-works/elsa-activity-sensing |
| 55 | Elsi Smart Floor | UNMT – Ambient Intelligence | https://www.maricare.com/en/how-it-works/elsi-smart-floor |
| 56 | Emoha | UNMT – Wearable | https://emoha.com/ |
| 57 | Etouch-bot | SAR Service Type | https://mobilerobotguide.com/2020/03/29/enova-robotics/ |
| 58 | Evondos | CMT | https://www.evondos.com/ |
| 59 | Fall Alert | CMT | https://personalalarms.org/products/suresafe-fall-alert |
| 60 | FallSafe | CMT | https://personalalarms.org/products/suresafe-fallsafe#features |
| 61 | Famil.care | UNMT – Wearable | https://famil.care/home?position=products |
| 62 | Folia Health | CMT | https://www.foliahealth.com/ |
| 63 | GAP | UNMT – Wearable | https://www.protom.com/2021/06/11/protom-la-digital-health-al-servizio-delle-rsa/ |
| 64 | Gigaset | UNMT – Ambient Intelligence | https://www.gigaset.com/it_it/cms/prodotti.html |
| 65 | GPS Smart Sole | UNMT – Wearable | https://gpssmartsole.com/gpssmartsole/ |
| 66 | GrandCare | UNMT – Ambient Intelligence | https://www.grandcare.com/ |
| 67 | Granny Vision | VRT | https://www.granny-vision.com/ |
| 68 | Health Data Analytics | UNMT – Wearable | http://reach2020.eu/?page_id=829 |
| 69 | HealthCam | UNMT – Ambient Intelligence | https://www.businesswire.com/news/home/20220106005082/en/Mitsubishi-Electric-Unveils-HealthCam-Scanning-Technology-that-Performs-Touchless-Line-of-Sight-Monitoring-of-Vitals |
| 70 | HealthyTogether | CMT | http://reach2020.eu/?p=4433 |
| 71 | Hector | SAR Service Type | https://www.robotcenter.co.uk/products/hector |
| 72 | HOBBIT | SAR Service Type | https://cordis.europa.eu/project/id/288146/it ; http://hobbit.acin.tuwien.ac.at/ |
| 73 | Home Wellness Kit | UNMT – Wearable & Ambient Intelligence | https://www.hinounou.com/Index/index/homecare |
| 74 | HomeCare | UNMT – Ambient Intelligence | https://www.develcoproducts.com/blog/facilitate-independent-living-among-seniors-with-home-care-kit/ |
| 75 | HomeGuardian | UNMT – Ambient Intelligence | https://homeguardian.ai/how-it-works/ |
| 76 | Human Activity Recognition | UNMT – Wearable & Ambient Intelligence | https://reach2020.eu/ |
| 77 | IBM MERA | SAR Service Type | https://www.ibm.com/blogs/research/2016/12/cognitive-assist/ |
| 78 | iGuard Stove | UNMT – Ambient Intelligence | https://iguardfire.com/ |
| 79 | Immerse Health | VRT | https://www.immersehealth.co.uk/90 |
| 80 | In Touch | UNMT – Wearable | https://bluebellbabymonitor.com/intouch |
| 81 | In-Home Classic Personal Alarm | CMT | https://taking.care/pages/personal-alarm-package |
| 82 | Independa Health Hub | CMT | https://independa.com/ |
| 83 | INSIEME | CMT | https://insieme.care/ |
| 84 | Isidora | CMT | https://cooplameridiana.it/nasce-loperatore-isidora/ |
| 85 | Joy for All | SAR Companion Type | https://joyforall.com/pages/our-story |
| 86 | Just Checking | UNMT – Ambient Intelligence | https://justchecking.co.uk/ |
| 87 | K4Community Smart Home | UNMT – Ambient Intelligence | https://www.k4connect.com/k4community-smart-home/ |
| 88 | Kaleido VR | VRT | https://kaleido.tours/ |
| 89 | Kanega | UNMT – Wearable | https://www.unaliwear.com/ |
| 90 | Kibi-companion | UNMT – Wearable & Ambient Intelligence | https://kibi.tech/come-funziona/ |
| 91 | Kibi Near | UNMT – Ambient Intelligence | https://kibi.tech/come-funziona/ |
| 92 | Kibi Wear | UNMT – Wearable | https://kibi.tech/come-funziona/ |
| 93 | Komp | CMT | https://www.noisolation.com/komp |
| 94 | Kompai | SAR Service Type | https://kompairobotics.com/ |
| 95 | Lean Empowering Assistant - LEA | SAR Service Type | http://www.robotikworld.com/lea/ |
| 96 | LHF - Connect | CMT | https://www.lhfconnect.net/ |
| 97 | Life Alert Emergency Button | CMT | https://www.lifealert.com/medical_50.aspx |
| 98 | Life Alert Help Button | CMT | https://www.lifealert.com/HELPButton.aspx |
| 99 | Life Alert Help On the Go + GPS | UNMT – Wearable | https://www.lifealert.com/HELPPhone_50.aspx |
| 100 | Life Alert Personal Protection at Home | CMT | https://www.lifealert.com/security_50.aspx |
| 101 | Lifeline with AutoAlert | UNMT – Wearable | https://www.lifeline.com/product/homesafe-with-autoalert-landline/ |
| 102 | LifePod | UNMT – Wearable | https://lifepod.com/ |
| 103 | LifeStation (In Home  Medical Alert System) | UNMT – Ambient Intelligence | https://www.lifestation.com/ |
| 104 | LISA habitec | UNMT – Ambient Intelligence + SAR Service Type | http://www.br2.ar.tum.de/wp-content/uploads/2021/03/1_Page_Summary_LISA_habitec.pdf |
| 105 | Lively Mobile Plus | UNMT – Wearable | www.mylively.com |
| 106 | Lively Wearable | UNMT – Wearable | https://www.lively.com/medical-alerts/lively-wearable2/ |
| 107 | LOOKY | UNMT – Ambient Intelligence | https://www.futurecare.it/ |
| 108 | LUNA Casa | UNMT – Ambient Intelligence | https://osatech.ch/telesoccorso/luna.html |
| 109 | LUNA Lights | UNMT – Ambient Intelligence | https://lunalights.org/ |
| 110 | MAGIC-GLASS | VRT | https://www.tech4care.it/magic-glass/ |
| 111 | MARIO | SAR Service Type | http://www.mario-project.eu/portal/ |
| 112 | MDSense | UNMT – Ambient Intelligence | https://www.essencesmartcare.com/ |
| 113 | Med-E-Lert | CMT | https://www.medelert.com/ |
| 114 | Medical Alert Basic | CMT | https://www.adt.com/health |
| 115 | Medical Alert Plus | UNMT – Wearable | https://www.adt.com/health |
| 116 | Medical Guardian (Home systems) | UNMT – Ambient Intelligence | https://www.medicalguardian.com/medical-alert-systems?filter=mobile |
| 117 | Medical Guardian (on-the-go systems) | UNMT – Wearable | https://www.medicalguardian.com/medical-alert-systems?filter=mobile |
| 118 | Medipad | UNMT – Wearable | https://osatech.ch/telesoccorso/chiamata-infermieri-allarmi-sensori-speciali.html |
| 119 | MedSign Qortext | CMT | https://www.medsign.com/medsign-makes-telehealth-simple-for-seniors/ |
| 120 | Mentorage | UNMT – Ambient Intelligence | https://wita.care/mentorage/ |
| 121 | MightyHealth | CMT | https://www.mightyhealth.com/ |
| 122 | Mindme Locate | UNMT – Wearable | https://taking.care/pages/gps-dementia-tracker |
| 123 | Mirana Bot | CMT | https://reach2020.eu/ |
| 124 | MiRo | SAR Companion Type | http://consequentialrobotics.com/miro-beta |
| 125 | Mobile Help (complete protection) | UNMT – Wearable | https://www.mobilehelp.com/collections/listed-products |
| 126 | Mobile Help (in home systems) | UNMT – Ambient Intelligence | https://www.mobilehelp.com/collections/listed-products |
| 127 | Mobile Help (on-the-go systems) | UNMT – Wearable | https://www.mobilehelp.com/collections/listed-products |
| 128 | MonAmi | UNMT – Wearable | https://www.monami.io/ |
| 129 | MoveCare | UNMT – Ambient Intelligence + SAR Service Type | http://www.movecare-project.eu/index.php/project/ |
| 130 | Mylo | SAR Service Type | https://www.heymylo.ie/ |
| 131 | mymedbook | UNMT – Wearable | https://www.mymedbook.eu/servizi-per-le-rsa/ |
| 132 | MyHelp Go | UNMT – Wearable | https://www.assistedlivingtechnologies.com/products/myhelp-go-1 |
| 133 | MyHelp In-Home Medical Alert Systems | UNMT – Ambient Intelligence | https://www.assistedlivingtechnologies.com/products/myhelp-landline-lte |
| 134 | MyHelp Teardrop Mobile | UNMT – Wearable | https://www.assistedlivingtechnologies.com/products/myhelp-go-1 |
| 135 | MYndVR | VRT | https://www.myndvr.com/ |
| 136 | MyNotifiRx® | UNMT – Wearable | https://medhab.com/mynotifirx/ |
| 137 | Nadine | SAR Companion Type | https://abcnews.go.com/Technology/human-robot-nadine-personality-mood-emotions-unveiled-singapore/story?id=36032196 |
| 138 | Near2U | UNMT – Wearable | https://liguria.bizjournal.it/2021/12/near2u-la-tecnologia-genovese-al-servizio-degli-anziani-per-migliorarne-sicurezza-e-assistenza/ |
| 139 | Neurotablet | CMT | https://www.neurab.com/neurotablet/ |
| 140 | Neurotrack | CMT | https://neurotrack.com/ |
| 141 | Nobi | UNMT – Ambient Intelligence | https://nobi.life/ |
| 142 | NoonCare | UNMT – Ambient Intelligence | https://noon.care/ |
| 143 | Nounou robot | SAR Service Type | https://www.hinounou.com/Index/index/homecare |
| 144 | On-The-Go | UNMT – Wearable | https://www.adt.com/health |
| 145 | Oroi | VRT | https://en.oroi.info/ |
| 146 | Oscar Senior | CMT | https://www.oscarsenior.com/ |
| 147 | Out-And-About Classic GPS Alarm | UNMT – Wearable | https://taking.care/pages/gps-personal-alarm |
| 148 | Out-And-About Mobile Alarm | UNMT – Wearable | https://taking.care/pages/oysta-gps-mobile-alarm |
| 149 | Out-And-About Personal Alarm Watch | UNMT – Wearable | https://taking.care/pages/personal-alarm-watch |
| 150 | Panic Button | UNMT – Wearable | https://www.develcoproducts.com/products/sensors-and-alarms/panic-button/ |
| 151 | Paro | SAR Companion Type | http://www.parorobots.com/index.asp |
| 152 | Path Feel | UNMT – Wearable | https://walkwithpath.com/pages/path-feel-coming-soon |
| 153 | Path Finder | UNMT – Wearable | https://walkwithpath.com/ |
| 154 | Pearl | SAR Service Type | https://www.researchgate.net/publication/2494731_Pearl_A_Mobile_Robotic_Assistant_for_the_Elderly |
| 155 | People Power Family | UNMT – Wearable | https://www.peoplepowerfamily.com/ |
| 156 | Pepper | SAR Companion Type | https://www.softbankrobotics.com/emea/en/pepper |
| 157 | PI2Bed | UNMT – Ambient Intelligence | http://reach2020.eu/ |
| 158 | PI2U-MiniArc | UNMT – Ambient Intelligence | http://reach2020.eu/ |
| 159 | PPP Taking Care | UNMT – Wearable | https://taking.care |
| 160 | Premier | UNMT – Wearable | https://electroniccaregiver.com/ |
| 161 | Pria | CMT | https://www.okpria.com/ |
| 162 | Pro Health | UNMT – Wearable | https://electroniccaregiver.com/ |
| 163 | QuietCare | UNMT – Ambient Intelligence | https://www.careinnovations.com/quietcare/ |
| 164 | Reminder Rosie (Talking Clock) | CMT | https://www.amazon.com/Reminder-Personalized-Reminders-Messages-Activated/dp/B09DJXXDZL?th=1 |
| 165 | Rendever | VRT | https://www.rendever.com/about/ |
| 166 | Robear | SAR Service Type | https://www.theguardian.com/technology/2015/feb/27/robear-bear-shaped-nursing-care-robot |
| 167 | Robot R1 | SAR Service Type | https://www.iit.it/web/icub/products/r1-robot |
| 168 | Rilevatore cadute (fall detector) | UNMT – Wearable | https://osatech.ch/telesoccorso/telesoccorso-avviso-caduta.html |
| 169 | Romeo | SAR Service Type | https://www.maxongroup.com/maxon/view/application/Romeo-A-helpful-friend-for-the-future |
| 170 | Safely You | UNMT – Ambient Intelligence | https://www.safely-you.com/ |
| 171 | Salute a casa | CMT | https://www.i-tel.it/salute-a-casa |
| 172 | Samsung Bot Care | SAR Companion Type & Service Type | https://news.samsung.com/global/video-meet-the-samsung-bots-your-companions-of-the-future |
| 173 | Samsung Bot Handy | SAR Service Type | https://news.samsung.com/it/better-normal-for-all-samsung-presenta-al-ces-le-innovazioni-2021-per-un-futuro-migliore |
| 174 | Sara | SAR Service Type | https://sara-robotics.com/en/home-3/ |
| 175 | SecureSeniorConnections | UNMT – Wearable | https://www.secureseniorconnections.com/ |
| 176 | Senior Living Safety System | UNMT – Wearable | https://healthcare.bestbuy.com/site/bbhealth/products-technology/pcmcat1600181550900.c?id=pcmcat1600181550900#jump-link-nav-header-3c550a82-8fde-4014-a451-88f319f19ea1 |
| 177 | SensorNet | UNMT – Ambient Intelligence | https://www.spazio50.org/assistenza-domiciliare-ecco-la-rete-intelligente-per-anziani-e-caregiver/ |
| 178 | Sensi.AI | UNMT – Ambient Intelligence | https://sensi.ai/ |
| 179 | SEREMY | UNMT – Wearable | https://www.seremy.it/ |
| 180 | Sengled Smart Bulbs | UNMT – Ambient Intelligence | https://it.sengled.com/it/ |
| 181 | SeniorVR | VRT | https://www.seniorvr.it/it |
| 182 | SkyAngelCare | UNMT – Wearable | https://www.amazon.com/SkyAngelCare-Detection-Compatible-Together-Automatically/dp/B09LGJYPY8 |
| 183 | SmartCare | UNMT – Ambient Intelligence | https://ec.europa.eu/regional_policy/it/projects/Italy/smartcare-using-ict-to-enable-older-people-to-live-independently-for-longer |
| 184 | Smart Life in Fife | UNMT – Wearable | https://www.smartlifeinfife.org/Home/About |
| 185 | SOFIHUB Beacon | UNMT – Wearable | https://www.sofihub.com/2020/12/13/safety-pendant-for-seniors/ |
| 186 | SOFIHUB eazense - Fall detection monitor | UNMT – Ambient Intelligence | https://www.sofihub.com/eazense/ |
| 187 | SOFIHUB TEQ-Home | UNMT – Ambient Intelligence | https://www.sofihub.com/sofihub-home-4-2/ |
| 188 | StackCare | UNMT – Ambient Intelligence | https://stack.care/ |
| 189 | SureSafeGO 24/7 Connect | UNMT – Ambient Intelligence | https://personalalarms.org/products/suresafego#features |
| 190 | SureSafe Guardian | CMT | https://personalalarms.org/products/suresafe-guardian#features |
| 191 | SureSafe Guardian Plus | UNMT – Wearable | https://personalalarms.org/products/suresafe-guardian#features |
| 192 | Tahoma 2.0 | UNMT – Ambient Intelligence | https://www.somfy.it/somfy-blog/somfy-magazine/articolo/domotica-assistenziale-per-vivere-meglio-nella-propria-casa |
| 193 | Taking Care Anywhere | UNMT – Wearable | https://taking.care/pages/taking-care-anywhere |
| 194 | Taking Care Safe Home Alert | UNMT – Wearable | https://taking.care/pages/taking-care-safe-home-alert |
| 195 | Taking Care Sense | UNMT – Wearable | https://taking.care/pages/taking-care-sense-home-monitoring#:~:text=Taking%20Care%20Sense,-From%20%C2%A39.99&text=A%20proactive%20home%20monitoring%20system,solution%20to%20provide%20care%20support. |
| 196 | TalkSafe | CMT | https://personalalarms.org/products/suresafe-talksafe |
| 197 | TalkToPoppy! | UNMT – Ambient Intelligence | https://www.talktopoppy.com/ |
| 198 | Tellus You Care | UNMT – Wearable | www.tellusyoucare.com |
| 199 | temi | SAR Service Type | https://www.robotemi.com/ |
| 200 | Ti-Seguo | UNMT – Wearable | https://osatech.ch/telesoccorso/chiamata-infermieri-tentativi-di-fuga.html |
| 201 | Tiago Robot | SAR Service Type | https://pal-robotics.com/robots/tiago/ |
| 202 | TOMBOT | SAR Companion Type | https://tombot.com/ |
| 203 | TrueLoo | UNMT – Ambient Intelligence | https://www.toilabs.com/trueloo/ |
| 204 | TurnTable | UNMT – Ambient Intelligence | https://www.unica.it/unica/page/it/turntable_tecnologia_e_ricerca_scientifica_per_il_benessere_degli_anziani |
| 205 | VarioSwitch | UNMT – Ambient Intelligence | https://osatech.ch/telesoccorso/chiamata-infermieri-allarmi-sensori-speciali.html |
| 206 | VayyarCare | UNMT – Ambient Intelligence | https://vayyar.com/care/ |
| 207 | Visavis | CMT | https://wita.care/visavis/ |
| 208 | VitalBand | UNMT – Wearable | https://www.amazon.co.uk/VitalBAND-Activity-RESISTANT-automatic-detection/dp/B07DFPVCSK |
| 209 | Vitalerter | UNMT – Ambient Intelligence | https://www.artech-srl.com/vitalerter/ |
| 210 | V-SOS Band | UNMT – Wearable | https://eshop.v.vodafone.com/it/v-sos-band |
| 211 | Walabot Home | UNMT – Ambient Intelligence | https://apps.apple.com/it/app/walabot-home-fall-detection/id1437493063 |
| 212 | Walk Wise | UNMT – Wearable | www.walkwise.com |
| 213 | Watchseniors | UNMT – Wearable | https://watchseniors-it.com/ |
| 214 | Wearable Health Monitoring System | UNMT – Wearable | http://reach2020.eu/?page_id=953 |
| 215 | Weenect | UNMT – Wearable | https://www.weenect.com/it/salvavita-anziani/ |
| 216 | WIISEL | UNMT – Wearable | https://www.inrca.it/INRCA/MODM2/ |
| 217 | WiMBeds | UNMT – Ambient Intelligence | https://www.wimonitor.it/wimonitor/it/ |
| 218 | WiMDoor | UNMT – Ambient Intelligence | https://www.wimonitor.it/wimonitor/it/ |
| 219 | WiMHome | UNMT – Ambient Intelligence | https://www.wimonitor.it/wimonitor/it/ |
| 220 | Zembro Plus | UNMT – Wearable | https://osatech.ch/telesoccorso/telesoccorso-gps-zembro.html |
| 221 | Zibrio | UNMT – Ambient Intelligence | https://www.zibrio.com/ |
| 222 | ZORA | SAR Service Type | https://www.robotlab.com/store/zora-robot-solution-for-healthcare |
